# Supplementary material for: Structure-activity relationships of mitochondria-targeted tetrapeptide pharmacological compounds
Source: eLife. 2022 Aug 1;11:e75531. doi: 10.7554/eLife.75531 (PMC9342957; doi:10.7554/eLife.75531)
Supplement: Supplementary file 1. [file elife-75531-supp1.docx]

| **SPN10** | **H** | **N** | **Hα** | **Cα** | **Hβ** | **Cβ** | **other** |
| --- | --- | --- | --- | --- | --- | --- | --- |
| Trp1 | - | - | 4.16 | 57.02 | 3.13 | 30.24 | Hδ1 7.06; Hε1 9.99; Hε3 7.44; Hζ2 7.39; Hζ3 7.01; Hη2 7.13; Cδ1 128.61; Cε3 121.21; Cζ2 115.27;  Cζ3 122.73; Cη2 125.34; Nε1 130.32 |
| Arg2 | 8.15 | 123.39 | 4.14 | 56.79 | 1.48, 1.54 | 32.00 | Hγ 1.32; Hδ 2.99; Hsc 6.90, 7.05; Cγ 27.47;  Cδ 43.86, Nε 96.86 |
| Trp3 | 8.01 | 123.13 | 4.43 | 57.62 | 3.12 | 30.16 | Hδ1 7.16; Hε1 10.09; Hε3 7.56; Hζ2 7.33; Hζ3 7.08; Hη2 7.16; Cδ1 127.99; Cε3 121.52; Cζ2 115.34;  Cζ3 122.75; Cη2 125.37; Nε1 129.51 |
| Lys4 | 7.96 | 124.35 | 4.00 | 56.80 | 1.48, 1.59 | 33.68 | Hγ 1.14, Hδ 1.48; Hε 2.78; Cγ 25.01; Cδ 29.87;  Cε 42.61 |
| C’NH_2_ | 6.67, 6.77 | 107.45 |  |  |  |  |  |
| **SS-20** | **H** | **N** | **Hα** | **Cα** | **Hβ** | **Cβ** | **other** |
| Phe1 | - | - | 4.11 | 57.45 | 3.05, 3.24 | 40.36 | Hδ 7.25; Hε 7.33; Hζ 7.29; Cδ 132.00; Cε 130.59; Cζ 130.07 |
| *arg2* | 7.06 | N.D. | 4.15 | 56.16 | 1.16, 1.20 | 30.63 | Hγ 0.72, 0.76; Hδ 2.83; Hsc 6.90, 7.13;Cγ 26.61;  Cδ 43.11; Nε 96.80 |
| Phe3 | 8.75 | 122.21 | 4.74 | 57.51 | 2.92, 3.28 | 39.80 | Hδ 7.33; Hε 7.36; Hζ 7.42; Cδ 131.90; Cε 131.62; Cζ 131.83 |
| Lys4 | 8.45 | 123.48 | 4.30 | 56.17 | 1.79, 1.87 | 33.08 | Hγ 1.43, 1.48; Hδ1.67,1.71; Hε 2.99; Cγ 24.92;  Cδ 29.10; Cε 41.88 |
| C’NH_2_ | 7.22, 7.43 | 108.63 |  |  |  |  |  |
| **SPN4** | **H** | **N** | **Hα** | **Cα** | **Hβ** | **Cβ** | **other** |
| *arg1* | - | - | 3.96 | 54.35 | 1.66 | 29.84 | Hγ 1.11, 1.12; Hδ 3.01; Cγ 24.19; Cδ 41.79 |
| Tyr2 | 8.86 | 118.64 | 4.73 | N.D. | 2.76, 3.08 | 37.87 | Hδ 7.18; Hε 6.87; Cδ 131.97; Cε 117.02 |
| Lys3 | 8.52 | 123.10 | 4.33 | 55.02 | 1.74, 1.67 | 31.94 | Hγ 1.40, 1.34; Hδ 1.66; Hε 3.00; Cγ 23.21; Cδ 27.85; Cε 40.85 |
| Phe 4 | 8.30 | 122.15 | 4.65 | 55.75 | 3.08, 3.21 | 38.37 | Hδ 7.36; Hε 7.38; Hζ 7.30; Cδ 130.74; Cε 130.22; Cζ 128.83 |
| C’NH_2_ | 7.16, 7.65 | 109.26 |  |  |  |  |  |
| **SS-31** | **H** | **N** | **Hα** | **Cα** | **Hβ** | **Cβ** | **other** |
| *arg1* | - | - | 3.89 | 55.34 | 1.69 | 31.21 | Hγ 1.29; Hδ 3.13; Cγ 25.55; Cδ 43.09 |
| Tyx2 | 8.81 | 122.40 | 4.75 | 61.92 | 2.96, 3.17 | 33.17 | Hδ 2.24; Hε 6.56; Cδ 21.72; Cε 117.28 |
| Lys3 | 8.08 | 124.01 | 4.32 | 56.00 | 1.71, 1.66 | 33.12 | Hγ 1.27, 1.33; Hδ 1.65; Hε 2.97; Cγ 23.96; Cδ 28.82; Cε 41.78 |
| Phe4 | 8.36 | 122.16 | 4.58 | 57.10 | 3.07, 3.17 | 39.24 | Hδ 7.41; Hε 7.35; Hζ 7.31; Cδ 131.16; Cε 131.48; Cζ 129.59 |
| C’NH_2_ | 7.16, 7.65 | 109.26 |  |  |  |  |  |

*^a^*NMR data were collected on 10 mM peptide samples, at pH 6, and a temperature of 25°C. The samples contained no added buffers or salts. Chemical shifts were referenced using 3-(Trimethylsilyl)propane-1-sulfonate (DSS) and are given in units of ppm. Because the free and bicelle-bound peptides are in fast exchange, and since there is an excess of the peptides, the chemical shift assignments in this table are also valid for the bound peptides.

*^b^*Underlined ^1^H chemical shifts show significant differences of ~0.4 ppm from the random coil values reported in Table 2.3 of Wüthrich, K. NMR of Proteins and Nucleic Acids. (John Wiley & Sons, New York; 1986). These upfield shifts are characteristic of aromatic ring current effects, and likely reflect basic residues involved in cation-pi interactions with aromatic rings.
