# Supplementary material for: Structure-activity relationships of mitochondria-targeted tetrapeptide pharmacological compounds
Source: eLife. 2022 Aug 1;11:e75531. doi: 10.7554/eLife.75531 (PMC9342957; doi:10.7554/eLife.75531)
Supplement: Supplementary file 2. [file elife-75531-supp2.docx]

| **NMR Structure** | **SPN10** | **SS-20** | **SPN4** | **SS-31** |
| --- | --- | --- | --- | --- |
| **Restraints** |  |  |  |  |
| NOEs (total) | 39 | 52 | 46 | 29 |
| Intraresidue NOEs | 28 | 33 | 33 | 14 |
| Sequential NOEs | 11 | 19 | 10 | 12 |
| NOEs( \|i-j\| = 2) | 0 | 0 | 3 | 3 |
| NOEs (\|i-j\| = 3) | 0 | 0 | 0 | 0 |
| Dihedral (φ,ψ)*^a^* | 4 | 4 | 4 | 4 |
| **Restraint Violations***^b,c^* |  |  |  |  |
| NOE (Å) | 0.0825 ± 0.0049 | 0.0523 ± 0.0030 | 0.00026 ± 0.00028 | 0.0422 ± 0.0109 |
| Dihedral (^o^) | 0.06 ± 0.15 | 0.0 ± 0.0 | 0.62 ± 0.20 | 0.12 ± 0.48 |
| **RMSD Ideal Geometry** |  |  |  |  |
| Bonds (Å) | 0.0036 ± 0.0003 | 0.0036 ± 0.0004 | 0.0042 ± 0.0002 | 0.0015 ± 0.0004 |
| Angles (^o^) | 0.35 ± 0.02 | 0.40 ± 0.03 | 0.46 ± 0.02 | 0.31 ± 0.04 |
| Impropers (^o^) | 0.17 ± 0.02 | 0.28 ± 0.04 | 0.26 ± 0.04 | 0.16 ± 0.03 |
| **Ramachandran Statistics***^d^* |  |  |  |  |
| Most favored (%) | 0 | 0 | 0 | 0 |
| Allowed (%) | 100 | 50 | 100 | 100 |
| Generously allowed (%) | 0 | 0 | 0 | 0 |
| Disallowed (%) | 0 | 50*^e^* | 0 | 0 |
| **Coordinate RMSD (Å)** *^f^* |  |  |  |  |
| Backbone (Cα+N+C’+O) | 1.32 ± 0.23 | 0.70 ± 0.15 | 0.56 ± 0.07 | 0.64 ± 0.24 |
| Heavy | 2.41 ± 0.59 | 1.62 ± 0.34 | 1.47 ± 0.34 | 1.16 ± 0.20 |

*^a^*Loose dihedral restraints of φ = -90 ± 70 and ψ = +60 ± 120 deg were included for the central residues 2 and 3 of the tetrapeptides. For the SS-20 peptide which has a D-arg at position 2, the φ restraint was set to +90 ± 70 deg.

*^b^*Values are given as the NMR ensemble mean ± SD

*^c^*Structures contained no distance violations greater 0.3 Å or dihedral violations greater than 5 degrees.

*^d^*Dihedral angles are only defined for the central residues 2 and 3 of the tetrapeptides.

*^e^*The SS-20 peptide has a D-arg at position 2, and its dihedral angles occur in the left-handed region of Ramachandran space.

*^f^*RMSD values were calculated using the GROMACS *gmx rms* function.
