# Supplementary material for: Structure-activity relationships of mitochondria-targeted tetrapeptide pharmacological compounds
Source: eLife. 2022 Aug 1;11:e75531. doi: 10.7554/eLife.75531 (PMC9342957; doi:10.7554/eLife.75531)
Supplement: Supplementary file 3. [file elife-75531-supp3.docx]

| **NMR Structure** | **SPN10** | **SS-20** | **SPN4** | **SS-31** |
| --- | --- | --- | --- | --- |
| **Restraints** |  |  |  |  |
| NOEs (total) | 95 | 113 | 100 | 106 |
| Intraresidue NOEs | 37 | 56 | 46 | 35 |
| Sequential NOEs | 47 | 39 | 43 | 55 |
| NOEs( \|i-j\| = 2) | 7 | 3 | 11 | 16 |
| NOEs (\|i-j\| = 3) | 4 | 15 | 0 | 0 |
| Dihedral (φ,ψ)*^a^* | 4 | 4 | 4 | 4 |
| **Restraint Violations***^b,c^* |  |  |  |  |
| NOE (Å) | 0.0720 ± 0.0018 | 0.0896 ± 0.0010 | 0.0697 ± 0.0019 | 0.0825± 0.003 |
| Dihedral (^o^) | 0.76 ± 0.36 | 1.76 ± 0.18 | 0.63 ± 0.19 | 1.14 ± 0.23 |
| **RMSD Ideal Geometry** |  |  |  |  |
| Bonds (Å) | 0.0078 ± 0.0002 | 0.0084 ± 0.0001 | 0.0056 ± 0.0002 | 0.0106 ± 0.0002 |
| Angles (^o^) | 0.97 ± 0.01 | 0.89 ± 0.02 | 0.56 ± 0.02 | 0.90 ± 0.03 |
| Impropers (^o^) | 0.65 ± 0.06 | 0.71 ± 0.03 | 0.34 ± 0.02 | 0.57 ± 0.04 |
| **Ramachandran Statistics***^d^* |  |  |  |  |
| Most favored (%) | 0 | 0 | 50 | 50 |
| Allowed (%) | 100 | 50 | 0 | 50 |
| Generously allowed (%) | 0 | 50*^e^* | 50 | 0 |
| Disallowed (%) | 0 | 0 | 0 | 0 |
| **Coordinate RMSD (Å)** *^f^* |  |  |  |  |
| Backbone (Cα+N+C’+O) | 0.06 ± 0.02 | 0.07 ± 0.15 | 0.04 ± 0.02 | 0.11 ± 0.03 |
| Heavy | 0.47 ± 0.09 | 0.55 ± 0.10 | 0.71 ± 0.19 | 0.90 ± 0.24 |

*^a^*Loose dihedral restraints of φ = -90 ± 70 and ψ = +60 ± 120 deg were included for the central residues 2 and 3 of the tetrapeptides. For the SS-20 peptide which has a D-arg at position 2, the φ restraint was set to +90 ± 70 deg.

*^b^*Values are given as the NMR ensemble mean ± SD

*^c^*Structures contained no distance violations greater 0.3 Å or dihedral violations greater than 5 degrees.

*^d^*Dihedral angles are only defined for the central residues 2 and 3 of the tetrapeptides.

*^e^*The SS-20 peptide has a D-arg at position 2, and its dihedral angles occur in the left-handed region of Ramachandran space.

*^f^*RMSD values were calculated using the GROMACS *gmx rms* function.
