# Supplementary material for: Structure-activity relationships of mitochondria-targeted tetrapeptide pharmacological compounds
Source: eLife. 2022 Aug 1;11:e75531. doi: 10.7554/eLife.75531 (PMC9342957; doi:10.7554/eLife.75531)
Supplement: Supplementary file 4. [file elife-75531-supp4.docx]

| **Ensemble Source** | **Heavy Atom RMSD (Å)** | | | | **Heavy Atom Radius of Gyration (Å)** | | | |
| --- | --- | --- | --- | --- | --- | --- | --- | --- |
|  | **SS-31** | **SS-20** | **SPN4** | **SPN10** | **SS-31** | **SS-20** | **SPN4** | **SPN10** |
| **NMR: Solution** | 1.16 ± 0.20 | 1.62 ± 0.34 | 1.47 ± 0.34 | 2.41 ± 0.59 | 4.57 ± 0.12 | 4.70 ± 0.13 | 4.72 ± 0.16 | 4.79 ± 0.20 |
| **NMR: Membrane** | 0.90 ± 0.24 | 0.55 ± 0.10 | 0.71 ± 0.19 | 0.47 ± 0.09 | 4.63 ± 0.18 | 4.20 ± 0.03 | 4.08 ± 0.08 | 4.23 ± 0.03 |
| **MD: Solution** | 2.39 ± 0.43 | 2.60 ± 0.15 | 2.22 ± 0.30 | 3.23 ± 0.12 | 5.38 ± 0.13 | 5.42 ± 0.08 | 5.44 ± 0.05 | 5.22 ± 0.13 |
| **MD: Membrane** | 1.89 ± 0.27 | 1.89 ± 0.43 | 2.04 ± 0.34 | 1.83 ± 0.42 | 5.78 ± 0.12 | 5.22 ± 0.10 | 5.67 ± 0.14 | 5.45 ± 0.17 |
| **MD: Membrane (restrained)** | 1.93 ± 0.28 | 2.07 ± 0.34 | 2.03 ± 0.29 | 1.44 ± 0.37 | 5.49 ± 0.10 | 4.86 ± 0.12 | 5.43 ± 0.12 | 5.08 ± 0.21 |
