# Supplementary material for: Structure-activity relationships of mitochondria-targeted tetrapeptide pharmacological compounds
Source: eLife. 2022 Aug 1;11:e75531. doi: 10.7554/eLife.75531 (PMC9342957; doi:10.7554/eLife.75531)
Supplement: Supplementary file 5. [file elife-75531-supp5.docx]

| **Peptide Analog** | **% Time <3Å Heavy Atom RMSD** | | | |
| --- | --- | --- | --- | --- |
|  | **Unrestrained** | | **w/ NOE Restraints** | |
| **SS-31** | **Avg.** | 5.70% | **Avg.** | 52.11% |
|  | **Std. Dev.** | 5.32% | **Std. Dev.** | 26.16% |
|  | **Range** | 0.89 - 17.78% | **Range** | 5.24 - 95.11% |
| **SS-20** | **Avg.** | 2.90% | **Avg.** | 20.27% |
|  | **Std. Dev.** | 3.75% | **Std. Dev.** | 29.71% |
|  | **Range** | 0.00 - 9.84% | **Range** | 0.01 - 94.79% |
| **SPN4** | **Avg.** | 1.95% | **Avg.** | 57.98% |
|  | **Std. Dev.** | 2.48% | **Std. Dev.** | 20.49% |
|  | **Range** | 0.19 - 11.28% | **Range** | 7.93 - 92.13% |
| **SPN10** | **Avg.** | 0.01% | **Avg.** | 32.10% |
|  | **Std. Dev.** | 0.02% | **Std. Dev.** | 36.31% |
|  | **Range** | 0.00 - 0.06% | **Range** | 0.00 - 97.66% |
