# Supplementary material for: Structure-activity relationships of mitochondria-targeted tetrapeptide pharmacological compounds
Source: eLife. 2022 Aug 1;11:e75531. doi: 10.7554/eLife.75531 (PMC9342957; doi:10.7554/eLife.75531)
Supplement: Supplementary file 6. [file elife-75531-supp6.docx]

| **Parameter measured in this study** | | **Similarities** | **Differences** | **Implications /**  **Outcomes** |
| --- | --- | --- | --- | --- |
| **Tetrapeptide**  **structure** | **Solution**  Figure 1B, C | • All peptides generally unstructured  • Some residual structure mediated by aromatic ring stacking and cation-π interactions | • Peptides with B-φ-B-φ register had some residual structure, mediated by aromatic residues | • Folded state promotes asymmetric charge distribution for peptide-lipid interactions at the polar-apolar boundary  • Engineering peptides structurally constrained to match bound state may improve potency |
|  | **Membrane-bound**  Figure 1C  Figure 3 | • Membrane binding generally causes peptides to become more compact and conformationally restrained | • SS-20, SPN4 and SPN10 form H-bonded reverse turn structures, whereas SS-31 is structurally extended |  |
| **Equilibrium binding to**  **CL-containing membranes** | **Binding affinity**  Figure 2  Figure 4 – supplement 1-5 | • All peptides have similar *K*_D_ (ITC)  • All peptides rapidly bind bilayers (MD) |  | • μM binding affinity much lower than typical (~nM) ligand-receptor drug interactions, consistent with interaction with fluid, dynamic lipid membrane  • Binding affinity (*K*_D_) does not strongly depend on side chain composition, but the lipid:peptide stoichiometry (*n*) and binding Δ*H* and Δ*S* do vary in a peptide-dependent manner  • Binding Δ*H* directly related to number of polar groups on aromatic side chains |
|  | **Binding density**  Figure 2 | • About 4-8 lipids per bound peptide | • SS-20 binds with a lower surface density  • SPN10 binds with a higher surface density |  |
|  | **Thermodynamic parameters**  Figure 2 | • Binding enthalpy is favorable (Δ*H*<0)  • Binding dominated by favorable entropy (TΔ*S*>0) | • SS-20 binding is more entropy-driven than SPN10  • SPN10 binding is more enthalpy-driven than SS-20 |  |
| **Peptide interaction**  **with membrane** | **Binding depth**  Figure 4 C,D  Figure 3 – supplement 3,4 | • Bound peptides reside in the interfacial region  • Peptide-lipid NOEs generally between aromatic side chains and lipid protons close to headgroup | • SS-20 and SPN4 bound more superficially  • SS-31 and SPN10 bound more deeply | • Peptide binding depth may cause differential effects on lateral pressure profiles at different points along the Z-axis of a bilayer  • SPN10 maintained the lowest total SASA (lower surface roughness), which could have implications for molecular interactions at the interface |
|  | **SASA**  Figure 4D | • All peptides reduced headgroup and acyl chain SASA, but to different extents |  |  |
| **Peptide effects on membrane properties** | **Bilayer thickness /**  **area per lipid**  Figure 4 – supplement 8 | • Binding of all peptides caused the expected inverse relationship between bilayer thickness and mean lipid area | • SS-31 and SPN10 expand area per lipid (decrease bilayer thickness) more than SS-20 and SPN4 | • Peptides that bound more deeply (SS-31 and SPN10) also caused greater expansion of membrane area / decrease of bilayer thickness  • Peptide effects on lipid-lipid interactions could have implications for lipid microdomain formation  • Reduced Ψ_s_ could modulate interactions of cations and polybasic proteins with CL-containing membranes and/or facilitate curvature by lowering anionic headgroup repulsion  • Altered Ψ_s_ could affect elastic properties of membranes and/or channel gating  • Tetrapeptides do not inherently cause depolarization or hyperpolarization of membranes |
|  | **Lateral peptide-lipid interaction**  Figure 3 – supplement 4  Figure 4 – supplement 9 | • Most peptide-lipid contacts through aromatic side chains and lipid regions close to headgroup | • SS-31 promotes CL self-interactions  • SPN10 minimizes CL self-interactions |  |
|  | **Surface potential (Ψ_s_)**  Figure 5 A,B | • All peptides down-regulate Ψ_s_ (all reduce surface charge) | • SPN10 attenuates Ψ_s_ much more strongly than other peptides |  |
|  | **Dipole potential (Ψ_d_)**  Figure 5C | • All peptides down-regulate Ψ_d_ (all disorder water/lipid dipoles) | • SS-20 attenuates Ψ_d_ much less than other peptides |  |
|  | **Transmembrane potential (ΔΨ_m_)**  Figure 5D | • No effect of any peptides on ΔΨ_m_ (all maintain transmembrane ion gradient) |  |  |
| **Peptide interaction with cells / protection against cell stress** | **Cell permeation and mitochondrial targeting**  Figure 6A | • Tested peptides (bio-SS-31 and bio-SPN10) permeate cells and localize to the mitochondrial network  • Partial restoration of membrane potential with serum deprivation provides strong evidence for a mitochondrial mode of action |  | • Requirements for cell permeation and mitochondria localization of tetrapeptides is highly promiscuous, needing only basic/aromatic R group content with no specific requirement for sequence register (B-φ-B-φ *vs*. φ-B-φ-B) or specific basic/aromatic side chains  • Rank ordering of ΔΨ_m_ restoration does not exactly mirror that of ATP content and cell viability; however, in all cases, SPN10 ranks highest |
|  | **Pharmacological activity in cell culture**  Figure 6B-D  Figure 6 – supplement 1 | • All peptides are pharmacologically active in cell culture  • Peptides improve ΔΨ_m_, extent of mitochondrial network, and ATP content in serum starvation models, consistent with a mechanism that directly targets mitochondrial function  • No peptides affected the viability of non-stressed cells | • Rank order of TMRM intensity (ΔΨ_m_ recovery) with serum withdrawal stress: SPN4<SS-31<SS-20<SPN10  • Rank order of ATP content with serum withdrawal stress: SS-31<SS-20=SPN4<SPN10  • Rank order of cell viability with serum withdrawal stress: SS-31=SS-20<SPN4<SPN10 |  |
