# Supplementary material for: Structure-activity relationships of mitochondria-targeted tetrapeptide pharmacological compounds
Source: eLife. 2022 Aug 1;11:e75531. doi: 10.7554/eLife.75531 (PMC9342957; doi:10.7554/eLife.75531)
Supplement: Transparent reporting form [file elife-75531-transrepform1.docx]

***eLife’s* transparent reporting form**

We encourage authors to provide detailed information *within their submission* to facilitate the interpretation and replication of experiments. Authors can upload supporting documentation to indicate the use of appropriate reporting guidelines for health-related research (see [EQUATOR Network](http://www.equator-network.org/%20)), life science research (see the [BioSharing Information Resource](https://biosharing.org/)), or the [ARRIVE guidelines](http://www.plosbiology.org/article/info:doi/10.1371/journal.pbio.1000412) for reporting work involving animal research. Where applicable, authors should refer to any relevant reporting standards documents in this form.

If you have any questions, please consult our Journal Policies and/or contact us: [editorial@elifesciences.org](mailto:editorial@elifesciences.org).

**Sample-size estimation**

- You should state whether an appropriate sample size was computed when the study was being designed
- You should state the statistical method of sample size computation and any required assumptions
- If no explicit power analysis was used, you should describe how you decided what sample (replicate) size (number) to use

Please outline where this information can be found within the submission (e.g., sections or figure legends), or explain why this information doesn’t apply to your submission:

Sample size, consisting of n independent replicates, is clearly stated in all figure legends (see Replicates section for experiment-specific definitions of independent reps). For empirical measurements, sample sizes were not statistically computed by power analyses *a priori*. Rather, the experimental design based on all techniques used an appropriate n value from published studies for the given approach. For most analyses from molecular dynamics time course trajectories, block averaging was used to estimate proper sample sizes (see Fig. S17).

**Replicates**

- You should report how often each experiment was performed
- You should include a definition of biological versus technical replication
- The data obtained should be provided and sufficient information should be provided to indicate the number of independent biological and/or technical replicates
- If you encountered any outliers, you should describe how these were handled
- Criteria for exclusion/inclusion of data should be clearly stated
- High-throughput sequence data should be uploaded before submission, with a private link for reviewers provided (these are available from both GEO and ArrayExpress)

Please outline where this information can be found within the submission (e.g., sections or figure legends), or explain why this information doesn’t apply to your submission:

**A. Replicates.** For each experiment type in this study, the number of times an experiment was performed is denoted by the n value, as denoted in the Materials and Methods section and explicitly stated in each figure legend. All investigators on this team define an “n” as an independent experiment; namely, one that is distinct from the others in the dataset (i.e., independently prepared and/or biologically distinct, depending on technique). Hence, the n value for each experiment type in this study allows for statistically rigorous analysis designed to capture true sample variation. Technical replicates (repeated measurements on the same sample), if used, were performed only to assay instrument consistency or technique reproducibility and are never counted as an independent n value. Because this study used a wide range of approaches, the number of replicates varied by technique as follows:

***Structural techniques.*** A total of 20 final NMR structures (low-energy conformers with no experimental restraint violations) were calculated for each peptide, whether free or membrane bound. This number is standard for NMR structures and should be sufficient to sample conformational space (typically the number of NMR structures should be half the number of amino acids in the sequence, assuming that a dipeptide is the relevant unit of protein structure).

***Molecular dynamics.*** For peptide measurements in the membrane systems, each peptide (out of 20 total) represented an independent sample. Due to the breadth of systems and computational cost of accurately modeling membranes in MD simulations, there were no additional replicates of simulations in this study.

***Biochemical/biophysical measurements.*** Calorimetric and spectral measurements with model membranes and isolated mitochondria all used a minimum of n=3 replicates. In specific cases when unexpected differences among samples were observed, we performed additional independent experiments to confirm the differences. This was the case for ITC data with SS-20 and SPN10 peptide analogs, which revealed noted differences in equilibrium binding parameters in comparison with the SS-31/SPN4 analogs.

***Cell culture studies.*** Studies on cellular ATP and cell viability were carried out in HK-2 or ARPE-19 cells grown in 96-well plates. All treatments were carried out with n=5 in each experiment. Results were normalized to the corresponding control group that received no treatment and a mean value was calculated for each treatment.

**B. Outliers.** As a general rule, outliers are determined as values possessing a Z-score greater than +3 or less than -3. All replicates for each peptide type have been included in this study and reported. The only exceptions were rare cases in ITC datasets when, likely due to technical issues, binding curves did not meet our criteria for saturation and/or calculated equilibrium binding parameters fit the exclusion criteria as outliers.

**Statistical reporting**

- Statistical analysis methods should be described and justified
- Raw data should be presented in figures whenever informative to do so (typically when N per group is less than 10)
- For each experiment, you should identify the statistical tests used, exact values of N, definitions of center, methods of multiple test correction, and dispersion and precision measures (e.g., mean, median, SD, SEM, confidence intervals; and, for the major substantive results, a measure of effect size (e.g., Pearson's r, Cohen's d)
- Report exact p-values wherever possible alongside the summary statistics and 95% confidence intervals. These should be reported for all key questions and not only when the p-value is less than 0.05.

Please outline where this information can be found within the submission (e.g., sections or figure legends), or explain why this information doesn’t apply to your submission:

Data reported in this study are commonly represented as means with variance calculated as SD, as clearly indicated in corresponding figure legends. The statistical analyses used to compare among sample groups are clearly described in the corresponding sections within the Materials and Methods section. For MD work, the non-parametric Wilcoxon-rank sum statistical test was used to test for statistical significance. For empirical work, differences among groups were evaluated by one-way ANOVA with post-hoc analyses carried out using Tukey’s multiple comparisons test. Comparison groups (e.g., control vs. treatment) for each experiment are clearly indicated in figure legends, as are all relevant *P* value classes.

(For large datasets, or papers with a very large number of statistical tests, you may upload a single table file with tests, Ns, etc., with reference to sections in the manuscript.)

**Group allocation**

- Indicate how samples were allocated into experimental groups (in the case of clinical studies, please specify allocation to treatment method); if randomization was used, please also state if restricted randomization was applied
- Indicate if masking was used during group allocation, data collection and/or data analysis

Please outline where this information can be found within the submission (e.g., sections or figure legends), or explain why this information doesn’t apply to your submission:

Group allocation does not apply to any of the experiments in this study. All samples (computational, reductionist model systems, or biological systems) were prepared for controlled comparisons among treatment with the four different peptide analogs.

**Additional data files (“source data”)**

- We encourage you to upload relevant additional data files, such as numerical data that are represented as a graph in a figure, or as a summary table
- Where provided, these should be in the most useful format, and they can be uploaded as “Source data” files linked to a main figure or table
- Include model definition files including the full list of parameters used
- Include code used for data analysis (e.g., R, MatLab)
- Avoid stating that data files are “available upon request”

Please indicate the figures or tables for which source data files have been provided:

Source data are provided as follows:

**Main text, Figure 1**: RMSD vs Rg scatter plot data of Fig 1C are compiled as Excel files and uploaded as Source Data linked to Fig. 1.

**Main text, Figure 2**: Data from binding curves (Q [raw data, means and SD], Fig. 2A) and summary of equilibrium binding parameters (raw data, means and SD, Fig. 2B) are compiled as Excel files and uploaded as Source Data linked to Fig. 2.

**Main text, Figure 4**: Data from chain-specific %Time <3Å RMSD +/- NOE restraints (Fig. 4B), membrane insertion depths (raw data, means and SD, Fig. 4C), and SASA analysis (raw data, means and SD, Fig. 4D) are compiled as Excel files and uploaded as Source Data linked to Fig. 4.

**Main text, Figure 5**: Data from ANS analyses (time courses and fractional saturation [raw data, means and SD] for model membranes and mitoplasts, Fig. 5A,B), ANEPPS analyses (ratiometric values [raw data, means and SD] and fractional saturation [raw data, means and SD], Fig. 5C) and TMRM analysis (time courses and fractional change [raw data, means and SD], Fig. 5D) are compiled as Excel files and uploaded as Source Data linked to Fig. 5.

**Main text, Figure 6**: Data from cell viability (raw data, means and SD, Fig. 6B) and cellular ATP (raw data, means and SD, Fig. 6C) are compiled as Excel files and uploaded as Source Data linked to Fig. 6.

**Additional data files**: Molecular dynamics input and data files are available at: <https://github.com/MayLab-UConn/SSpeptides_qsar_MDdata>.
